# Temperature effects on performance and physiology of two prairie stream minnows

**DOI:** 10.1093/conphys/coz063

**Published:** 2019-10-31

**Authors:** Bryan D Frenette, Lindsey A Bruckerhoff, Michael Tobler, Keith B Gido

**Affiliations:** Division of Biology, Kansas State University, 166 Ackert Hall, Manhattan, KS 66506, USA

**Keywords:** Activity, fish, metabolism, swimming performance, temperature, thermal limits

## Abstract

Earth’s atmosphere has warmed by ~1°C over the past century and continues to warm at an increasing rate. Effects of atmospheric warming are already visible in most major ecosystems and are evident across all levels of biological organization. Linking functional responses of individuals to temperature is critical for predicting responses of populations and communities to global climate change. The southern redbelly dace *Chrosomus erythrogaster* and the central stoneroller *Campostoma anomalum* are two minnows (Cyprinidae) that commonly occur in the Flint Hills region of the USA but show different patterns of occurrence, with dace largely occupying headwater reaches and stonerollers persisting in both headwater and intermediate-sized streams. We tested for differences between species in critical thermal maximum, energy metabolism, sustained swimming and activity over an ecologically relevant temperature gradient of acclimation temperatures. Typically, metrics increased with acclimation temperature for both species, although stoneroller activity decreased with temperature. We observed a significant interaction between species and temperature for critical thermal maxima, where stonerollers only had higher critical thermal maxima at the coldest temperature and at warm temperatures compared to the dace. We did not find evidence suggesting differences in the energy metabolism of dace and stonerollers. We detected interspecific differences in sustained swimming performance, with dace having higher swimming speed than stonerollers regardless of acclimation temperature. Finally, there was a significant interaction between temperature and species for activity; dace activity was higher at intermediate and warm temperatures compared to stonerollers. We observed subtle interspecific differences in how performance metrics responded to temperature that did not always align with observed patterns of distribution for these species. Thus, other ecological factors likely are important drivers of distributional patterns in these species.

## Teaser

The observed distributions of two grazing minnows differ along a stream-size gradient in grassland streams and may be linked to temperature. In laboratory experiments, we assayed a suite of physiological traits along a temperature gradient and found that species differed in critical thermal maxima, with subtle differences in other traits.

## Introduction

Temperature is a critical physical component of the environment that influences organisms across levels of biological organization (Addo-Bedaiko *et al*., 2000; Angilletta, 2009; Culumber *et al*., 2012; Jensen *et al*., 2017). Over the past century the earth’s atmosphere has warmed by ~1°C and continues to warm at an increasing rate, affecting ecosystems at global, regional and local scales (Walther *et al*., 2002; Angilletta, 2009; Eliason *et al*., 2011; Pachauri *et al*., 2014). Effects of climate change are already visible in most ecosystems, leading to shifts in the phenology of organisms, species ranges and community composition (Eaton and Scheller, 1996; Walther *et al*., 2002; Angilletta, 2009; Barceló *et al*., 2016). Understanding the role of temperature in shaping the performance of organisms will help researchers predict the impacts of climate warming on species’ distributions (Pörtner and Knust, 2007; Primack *et al*., 2009; Killen, 2014). A mechanistic understanding of the physiological processes that underlie an organism’s thermal biology will aid conservation efforts in the face of global change by bolstering the predictive ability of models of species distribution beyond the range of currently available environmental data (Teal *et al*., 2018).

The fundamental thermal niche of ectotherms, whose body temperatures are dependent on the environment, can be defined broadly as the range of body temperatures allowing for positive population growth (Magnuson *et al*., 1979; Gvoždík, 2018). Physiological mechanisms—including preferred body temperatures, thermal tolerances and energy metabolism—contribute to the fundamental thermal niche of an organism, as does the role of thermally related behaviour in influencing these mechanisms (Magnuson *et al*., 1979; Kearney and Porter, 2004, 2009; Gvoždík, 2018). The fundamental thermal niche is important in determining the entire range of temperatures over which populations of an organism can persist and is often included in species distribution models (Helaouët and Beaugrand, 2009). However, temperature is only one dimension of a species’ ecological niche and other components of the niche can also play a role in determining the temperature range where a species occurs. A species’ realized thermal niche may, therefore, represent a narrower range of temperatures after considering the influence of other biotic and abiotic factors (and their interactions with temperature) on the fundamental thermal niche (Gvoždík, 2018). Understanding the underlying mechanism driving thermal physiology is an important consideration when examining species’ distributions along temperature gradients, as well as their expected response to climate change (Grossman *et al*., 2010; Clark *et al*., 2013; Stoffels *et al*., 2016).

The distributions of fish species are expected to shift as climate change pushes water temperatures towards their upper thermal limits (Caissie, 2006). Grassland streams exist in one of the world’s most threatened biomes and understanding the thermal biology of grassland fishes will aid in predicting how populations of these species will respond to a changing climate (Dodds *et al*., 2004; Troia *et al*., 2016). In the Great Plains of North America, the biodiversity of stream fishes has declined over the past several decades, largely due to anthropogenic impacts (Perkin *et al*., 2017). These systems naturally experience severe fluctuations in environmental conditions due to cyclical patterns of drying and wetting, and fishes may experience thermal stress during periods of drought (Matthews, 1988; Dodds *et al*., 2004). Thermal stress might be exacerbated by fragmentation that inhibits dispersal into habitats with cooler water temperatures (Matthews and Zimmerman, 1990; Perkin *et al*., 2015). Under climate change scenarios, grassland streams are expected to be pushed towards more arid conditions and subjected to increases in the frequency and severity of droughts (Dodds *et al*., 2015). Physicochemical limitations associated with temperature are likely strong drivers of the distribution of fish species in prairie streams, and interspecific differences in thermal physiology or performance could facilitate replacement of species along a temperature gradient (Troia *et al*., 2016).

In prairie streams of the Flint Hills region in the central USA, cooler temperatures typically occur in spring-fed headwaters while downstream reaches are warmer (Troia and Gido, 2014; Troia *et al*., 2016). The southern redbelly dace (*Chrosomus erythrogaster*) and the central stoneroller (*Campostoma anomalum*) are two species of minnow (Cyprinidae) that occur in this region. Both species function in similar trophic roles, feeding primarily on algae (Bertrand and Gido, 2007). The critical thermal maximum (CTM) of the dace varies seasonally between ~17 and 32°C, while the stoneroller exhibits CTM as high as 38°C (Scott, 1987; Mundahl, 1990). Stoneroller and dace often co-occur in very small streams, but the occurrence of dace rapidly diminishes moving downstream, while stoneroller populations persist (Kansas Fishes Committee, 2014). Given the general pattern of increasing summer maximum temperature in downstream reaches (Troia and Gido, 2014), it is possible that dace are less tolerant of higher stream temperatures than stonerollers. Considering the apparent restriction of dace to headwater streams, the compounding effects of climate change and other anthropogenic disturbances may lead to the extirpation of dace populations from this region (Perkin *et al*., 2015, 2017). Understanding the mechanisms that contribute to the thermal niche of these species will aid in forecasting how they will respond to potential increases in temperature and thermal stress associated with drought.

Here we examined differences in physiological and performance responses over an ecologically relevant temperature gradient in the two species. Our general study aim was to test if dace and stoneroller differ in their physiological response to temperature, and therefore, differ in their fundamental thermal niches. We hypothesized that because stonerollers occur in larger, warmer streams, the response of physiological and performance metrics would be higher for stonerollers than for dace at warmer temperatures. We measured a suite of physiological and performance traits that allowed us to compare thermal responses in these species. These traits include CTM (the maximum temperature at which motor function ceases), aerobic metabolic scope (AMS), maximum sustained swimming speed (*U_crit_*; highest swimming velocity before fatigue) and activity (units of distance travelled per unit time).

## Materials and methods

### Fish distribution sampling

We collected dace and stonerollers from 188 sites in the Neosho and Kansas River basins in the Flint Hills ecoregion of eastern Kansas as part of another stream fish community survey. Sites were selected using a random-stratified design with the goal of capturing gradients of stream hierarchy (drainage basin and stream order) and catchment land use (percentage of cultivated lands and density of road crossings and impoundments). The watershed area of sites sampled ranged between <10 km^2^ and >200 km^2^. We sampled stream fish communities in the summer of 2017 in several pools and riffles (mesohabitats) at each site. Fish were sampled using single pass backpack electroshocking followed by multiple seine hauls (3 m × 1.2 m, 3.2 mm mesh seine) in all habitat types (large woody debris, vegetation, root wads, etc.) within each mesohabitat. We categorized streams into bins based on watershed area and visualized the abundances of both dace and stoneroller over this stream-size gradient.

### Characterizing stream temperatures

Submersible temperature loggers (Onset Corporation, Bourne, MA, USA) were placed in fixed locations at three stream sites—Kings Creek, McDowell Creek and Deep Creek—near Manhattan, KS. These three streams are tributaries to the Kansas River basin. Kings Creek is a low-order prairie stream found on the Konza Prairie Biological Station. McDowell Creek and Deep Creek are more intermediate-sized streams. Hourly temperature was recorded for each stream between 2013 and 2014 and was used to determine the mean daily temperature (°C ± standard deviation) and the coefficient of variation (%) of daily temperature for each stream.

### Experimental animals

For all experiments, dace and stonerollers were collected from the same ~ 30 m pool in Kings Creek near Manhattan, KS. Fishes were collected through a combination of seining (4.6 m × 1.8 m, 3.2 mm mesh seine) and direct current (DC)-pulsed backpack electrofishing (Smith-Root model 20B) and then transported to an aquarium facility at Kansas State University in large, aerated coolers (see Table 1 for the number and size range of individuals collected). We maintained a photoperiod of 12 h light: 12 h dark and a room temperature of 20°C in the aquarium facility. Fish were housed in either 38 or 76-L aquaria at a density of no more than 1 fish/6 L of water. Collections for CTM experiments occurred during the summer of 2015. For aerobic scope and critical swimming velocity experiments, fish were collected between March and August of 2017, and different fish were used in each experiment. Collections for activity experiments occurred in August of 2017.

**Table 1:** Mean (±1 SD) TL (mm), mean weight (g) and sample size (*N* = number of individuals) of dace and stonerollers in each temperature treatment used in CTM, metabolism, critical swimming velocity and activity experiments

Experiment	Species					
	Dace			Stoneroller		
	TL (mm)	Weight (g)	*N*	TL (mm)	Weight (g)	*N*
CTM						
7°C	35 (±2.1)	0.32 (±0.06)	10	53 (±6.2)	1.23 (±0.43)	10
12°C	36 (±3.0)	0.38 (±0.10)	10	53 (±5.2)	1.16 (±0.36)	10
17°C	37 (±6.1)	0.42 (±0.25)	11	53 (±6.9)	1.13 (±0.39)	9
22°C	35 (±2.3)	0.34 (±0.05)	8	52 (±5.1)	1.06 (±0.30)	10
27°C	36 (±3.3)	0.36 (±0.10)	10	62 (±6.7)	1.66 (±0.32)	6
Overall	36 (±3.7)	0.37 (±0.14)	49	54 (±6.6)	1.22 (±0.40)	45
Metabolism						
10°C	52 (±5.6)	1.28 (±0.43)	5	54 (±5.2)	1.33 (±0.26)	5
15°C	52 (±6.3)	1.29 (±0.52)	6	51 (±5.0)	1.19 (±0.37)	5
20°C	47 (±2.6)	0.88 (±0.14)	5	48 (±7.1)	1.03 (±0.40)	5
25°C	53 (±4.6)	1.33 (±0.32)	6	52 (±9.5)	1.31 (±0.64)	5
30°C	52 (±4.7)	1.31 (±0.33)	5	48 (±5.1)	0.83 (±0.08)	5
Overall	51 (±5.0)	1.22 (±0.38)	27	51 (±6.6)	1.14 (±0.41)	25
Critical swimming velocity						
10°C	54 (±7.7)	1.42 (±0.61)	8	55 (±8.6)	1.51 (±0.55)	8
15°C	55 (±6.0)	1.50 (±0.47)	8	58 (±10.0)	1.84 (±1.02)	8
20°C	53 (±9.5)	1.27 (±0.66)	8	59 (±7.9)	1.91 (±0.94)	8
25°C	55 (±6.4)	1.50 (±0.63)	8	51 (±9.5)	1.41 (±0.60)	8
30°C	55 (±9.1)	1.39 (±0.65)	8	55 (±6.4)	1.42 (±0.43)	8
Overall	54 (±7.5)	1.41 (±0.58)	40	56 (±8.6)	1.62 (±0.74)	40
Activity						
10°C	52 (±4.2)	1.03 (±0.24)	6	62 (±7.9)	1.89 (±0.70)	6
15°C	54 (±4.3)	1.29 (±0.46)	6	57 (±6.9)	1.43 (±0.34)	5
20°C	53 (±8.8)	1.16 (±0.52)	5	53 (±2.3)	1.22 (±0.15)	6
25°C	50 (±9.0)	0.95 (±0.61)	5	52 (±3.1)	1.09 (±0.18)	5
30°C	54 (±8.6)	1.11 (±0.55)	7	58 (±5.3)	1.37 (±0.35)	6
Overall	53 (±6.9)	1.11 (±0.47)	29	57 (±6.2)	1.41 (±0.47)	28

Rows highlighted in grey represent the mean values across all treatments in the experiment for length and weight and the total sample size for each species in the experiment.

Fish were held in the laboratory for 2 days at room temperature before being randomly assigned to an acclimation treatment appropriate to the experiment being conducted (treatments described in following sections). Either aquarium heaters or chillers were used to adjust treatment temperatures at a rate of 2°C/day until they reached the assigned treatment (Fangue *et al*., 2014). After reaching the treatment temperature, fish were acclimated for 14 days before beginning experiments—similar acclimation lengths have been used in other studies of fish thermal biology (McDonnell and Chapman, 2016; Malekar *et al*., 2018; Zhou *et al*., 2019). Unless otherwise noted, fish were fed a daily ration of commercial flake food and dried bloodworms. Fish were fasted for 48 h prior to their use in experiments to ensure a post-absorptive state (Allen-Ankins and Stoffels, 2017).

### CTM

We exposed dace and stonerollers to acute thermal stress to determine the CTM after acclimating them to different temperatures in the laboratory. For this experiment, we established five temperature acclimation treatments using separate 76-L tanks (7, 12, 17, 22 and 27°C) and randomly assigned 10 individuals of both species to each tank. These temperatures were chosen to represent possible mean ambient temperatures that occur across seasons in Kings Creek and other nearby grassland streams (Fig. 2). Subsequent experiments following this CTM experiment used a slightly different thermal gradient (10, 15, 20, 25 and 30°C) to more effectively utilize available heaters, chillers and aquarium space. Following the acclimation procedure described above, test fish were taken from their acclimation tanks and held in an aerated cooler that was kept at a constant temperature matching the acclimation temperature. Experimental trails were conducted on individual fish. Dechlorinated water was added to a 4 L testing chamber. A mesh cylinder was placed in the center of the chamber, and an air stone was placed inside the cylinder to oxygenate and mix the water, preventing a thermal gradient from developing. A mesh screen was placed over the chamber to prevent fish from escaping during a trial. Initial water temperature in the chamber was matched to the acclimation temperature being tested. The chamber was then placed on a heating plate, and one fish was added to the chamber. A HOBO logger (Onset Corporation, Bourne, MA, USA) configured to record temperature at 30 s intervals was immediately added to the chamber with the fish. The water in the chamber was heated at a rate of 0.3°C/min (Culumber *et al*., 2012; Chen *et al*., 2018). The temperature at which the fish experienced a loss of equilibrium was used to determine an individual’s CTM (Culumber *et al*., 2012; Culumber *et al*., 2014). Once a fish reached its CTM, the fish was removed and measured in millimeters total length (mm TL) and weighed in to the nearest 0.01 grams (g) (Table 1).

### Oxygen uptake and AMS

We used intermittent flow respirometry to measure mass-adjusted rates of oxygen consumption (MO_2_; mg O_2_ g^−1^ h^−1^) for both focal species along an acclimation gradient of temperature like the one described above (10, 15, 20, 25 and 30°C). We used as many as six dace and six stonerollers in each temperature treatment to measure standard metabolic rate (SMR), maximum metabolic rate (MMR) and AMS (total *n* = 52). We used a four-chamber intermittent flow respirometer (Loligo Systems, Tjele, Denmark) to measure metabolic rates following well-established methods (Crans *et al*., 2015; Svendsen *et al*., 2016). The volume of the respirometer chambers was 0.0987 L, and the relative mass of each fish to respirometer volume averaged ~0.01 g mL^−1^.

A submersible pump continuously circulated oxygenated water (≥95% O_2_ saturation) from a reservoir tank to the respirometer’s water bath. At the beginning of each week, we calibrated the respirometer’s optic oxygen probes following the manufacturer’s protocol. Dipping probe oxygen mini sensors were inserted into a probe vessel above a thin planar oxygen mini sensor that was housed inside the respirometry chambers (Loligo Systems, Tjele, Denmark). Either submersible heaters or a recirculating chiller was used to achieve a water temperature in the respirometer that matched the corresponding acclimation treatment. For each trial three fish were randomly drawn from an acclimation tank, and fish were randomly assigned to one of the four respirometry chambers. The remaining chamber was kept empty to serve as a control to measure background respiration during the trials (Jones *et al*., 2007; Svendsen *et al*., 2014; Rosewarne *et al*., 2016). Background respiration (mg O_2_ h^−1^) was calculated following equation (2) from Clark *et al*. (2013):

$$ \mathrm{M}{\mathrm{o}}_2=\frac{\left[\left({\mathrm{V}}_{\mathrm{t}}-{\mathrm{V}}_{\mathrm{f}}\right)\times \Delta{\mathrm{C}}_{\mathrm{w}{\mathrm{O}}_2}\right]}{\left(\Delta \mathrm{t}\times{\mathrm{M}}_{\mathrm{f}}\right)}, $$

where *V_t_* is the respirometer volume, *V_f_* is the fish volume and is assumed to be the same as fish mass (*M_f_*), *ΔC_wO2_* is the change in oxygen concentration in the respirometer water and *Δt* is the change in time during which the change in oxygen concentration is measured. Since the respirometer chamber used to measure background respiration is empty of fish, fish mass and fish volume are not included in the calculation (Rodgers *et al*., 2016). In our experiments, background respiration remained near zero (and <0.001% of fish respiration) across the 18 h experimental period.

Prior to introduction to the respirometry chamber, fish were measured in mm TL and weighed to the nearest 0.01 g (Table 1). The respirometer was programmed to operate with a 240 s flush phase (when oxygenated water is flushed into the chambers), a 60 s wait phase and a 240 s measure phase (modified from Stoffels, 2015). The respirometry phase cycles were controlled using AutoResp™ version 2 automated intermittent respirometry software (Loligo Systems, Tjele, Denmark). Fish were introduced to the respirometer chambers at 1400 h, and oxygen consumption rates were measured over the course of 18 h to allow fish to reach a constant resting state. The rate of oxygen consumption (MO_2_) was calculated from the slope of the regression of oxygen content (kPa) over time (h). We excluded the first 2 h of data to eliminate confounding effects of handling stress and used the average of the 10 lowest MO_2_ values collected over the remaining 16 h period to determine an individual’s SMR; MO_2_ values with an *r^2^* value of <0.9 were excluded.

Following SMR measurement, we employed a chase protocol to elicit MMR in individual fish (Brennan *et al*., 2016; Rosewarne *et al*., 2016). One fish was moved from the respirometry chamber to a circular arena containing aerated water with temperature matching the treatment temperature being tested. Using the handle of an aquarium dip net, the fish was chased in the arena until the fish exhibited burst-glide swimming and was considered exhausted when it would no longer respond to physical stimuli (in all trials, minimum chase time was 6 min). Following the chase protocol, fish were immediately reintroduced to the respirometer for the quantification of MMR (Rosewarne *et al*., 2016). We used the highest value of three cycles of the measure phase (240 s) to estimate MMR. This procedure was then repeated for the fish remaining in the respirometer, and all fish were tested between 0800 and 0900 h.

### Critical swimming velocity

We used a 5-L swim tunnel respirometer (Loligo Systems, Tjele, Denmark) to carry out swimming performance trials (Svendsen *et al*., 2015). We included eight dace and eight stonerollers in each of five temperature treatments (10, 15, 20, 25 and 30°C; total *n* = 80). We used either submersible heaters or ice packs to maintain water temperature in the swim tunnel within 0.2°C of the temperature treatment being tested. Trials of critical swimming velocity (*U*_crit_) were conducted using a velocity increment based on the total length (TL) of the fish (Brett, 1964; Morozov *et al*., 2018). Before being introduced to the swim chamber, individual fish were measured in mm TL and weighed to the nearest 0.01 g (Table 1). To discourage the fish from attempting to rest against the back screen of the swim chamber, we placed a partitioning shade over the front half of the chamber and shined light at the back of the chamber. Following methods for other fish species, fish were first acclimated to the chamber with no flow velocity for 30 min (Lehman *et al*., 2017) and then for 10 min at a water velocity of 1 TL s^−1^ before beginning the experiment. Swimming trials began at a water velocity of 2 TL s^−1^, with velocity being increased by 1 increment (1 TL) every 5 min and ending once the fish was fatigued (Nelson *et al*., 2003). Fatigue was determined as the velocity at which the fish became pinned to the back of the swim tunnel and would not return to swimming after visual or acoustic stimuli. The end of a dip net was used to prod the back of the swim chamber to stimulate the fish. *U*_crit_ was calculated in cm s^−1^ as

$$ \mathrm{Ucrit}=\mathrm{Ui}+\mathrm{Uii}\ \left(\frac{\mathrm{Ti}}{\mathrm{Ti}\mathrm{i}}\right), $$

where *U*_i_ is the highest velocity maintained for an entire 5 min interval, *U*_ii_ is the velocity increment, *T*_i_ is the time elapsed at fatigue velocity and *T*_ii_ is the time between velocity increments (Brett, 1964; Sfakuanakis *et al*., 2011). We calculated relative *U*_crit_ (*RU*_crit_) by dividing *U*_crit_ by body size (TL) to compare the swimming velocity corrected for body size (Brett, 1964; Gregory and Wood, 1998).

### Activity

For activity trials, we used six dace and six stonerollers for each temperature treatment (10, 15, 20, 25 and 30°C). Individual fish were placed into 27.5 × 17.5 cm plastic aquaria (hereafter, ‘arenas’) housed in a recirculating water bath after they were measured in mm TL and weighed to the nearest 0.01 g (Table 1). To prevent fish from seeing other individuals during the trail, the sides of the arenas were painted black. A recirculating chiller or submersible heater was used to maintain the water temperature in the water bath appropriate for the temperature treatment being tested. Each arena was kept aerated while housing fish. Arenas were placed over a 2 cm × 2 cm grid on a white background. Fish were habituated to the arenas for 7 days and fed a ration of frozen bloodworms each day during this period. Fish were fasted for 48 h before we stationed a GoPro® camera above each individual arena, capturing the entire arena in the field of view. The aerator was removed from the arena during video recording to prevent agitation of the water’s surface. Fish were recorded continuously for 15 min (Gautrias *et al*., 2009; Troia *et al*., 2016). We discarded the first 5 min of video to account for fish potentially being disturbed when the camera was turned on. Activity was quantified from the subsequent 10 min of video by randomly selecting a subsample of 10 non-overlapping 30-s intervals during the filmed trail and calculating the average number of 2 cm × 2 cm grids the fish traversed per unit time. All 12 fish belonging to a single acclimation temperature were filmed on the same day, beginning at 0900 h and finishing within 2 h. During filming trials, we recorded two fish concurrently using different cameras. Filming for all five acclimation treatments was completed in 5 days.

### Statistical analyses

We used two-way analysis of covariance (ANCOVA) to test for differences in CTM. We included acclimation temperature (treatment) and species as fixed factors and body size (mm TL) as a covariate (Angilletta, 2006; Allen-Ankins and Stoffels, 2017). Post hoc independent samples *t*-tests with a Bonferroni corrected significance threshold of α = 0.01 were conducted to compare the CTM of dace and stonerollers in each temperature treatment. We calculated AMS for an individual as the difference between its MMR and SMR. Data for SMR, MMR and AMS were analysed using two-way analysis of variance (ANOVA) and included acclimation temperature (treatment) and species as fixed factors. Metabolism data were ln-transformed prior to analysis to meet assumptions of normality. Since we collected and housed fish in the laboratory over different time blocks in some experiments, we first used a linear mixed-effects model and included sampling block as a random factor to account for any confounding effects. All species and acclimation treatments were represented in each sampling block. However, we found no significant effect of sampling block and were able to eliminate this parameter. We did not detect a significant temperature × species interaction for SMR (*F*_4,42_ = 1.679, *P* < 0.173), MMR (*F*_4,42_ = 1.135, *P* < 0.353) or AMS (*F*_4,42_ = 1.523, *P* < 0.213) and subsequently removed this interaction term. Tukey post hoc tests were used to determine differences between temperature treatments. Data for *RU*_crit_ were analysed using two-way ANOVA as described above and was ln-transformed prior to analysis to meet assumptions of normality. We did not detect a significant temperature × species interaction for *RU*_crit_ (*F*_4,70_ = 0.349, *P* < 0.844) and subsequently removed this interaction term. Post hoc independent samples *t*-tests with a Bonferroni corrected significance threshold of α = 0.01 were conducted to compare the *RU*_crit_ of dace and stonerollers in each temperature treatment. Tukey post hoc tests were used to determine differences between temperature treatments. We used two-way ANCOVA to test for differences in activity. We included acclimation temperature (treatment) and species as fixed factors and body size (mm TL) as a covariate. Data for activity were square-root-transformed prior to analysis to meet assumptions of normality. Body size did not have a significant effect on activity (*F*_1,46_ = 2.432, *P* = 0.126) and was removed from the analysis.

## Results

### Fish abundances along a stream-size gradient

**Figure 1:**
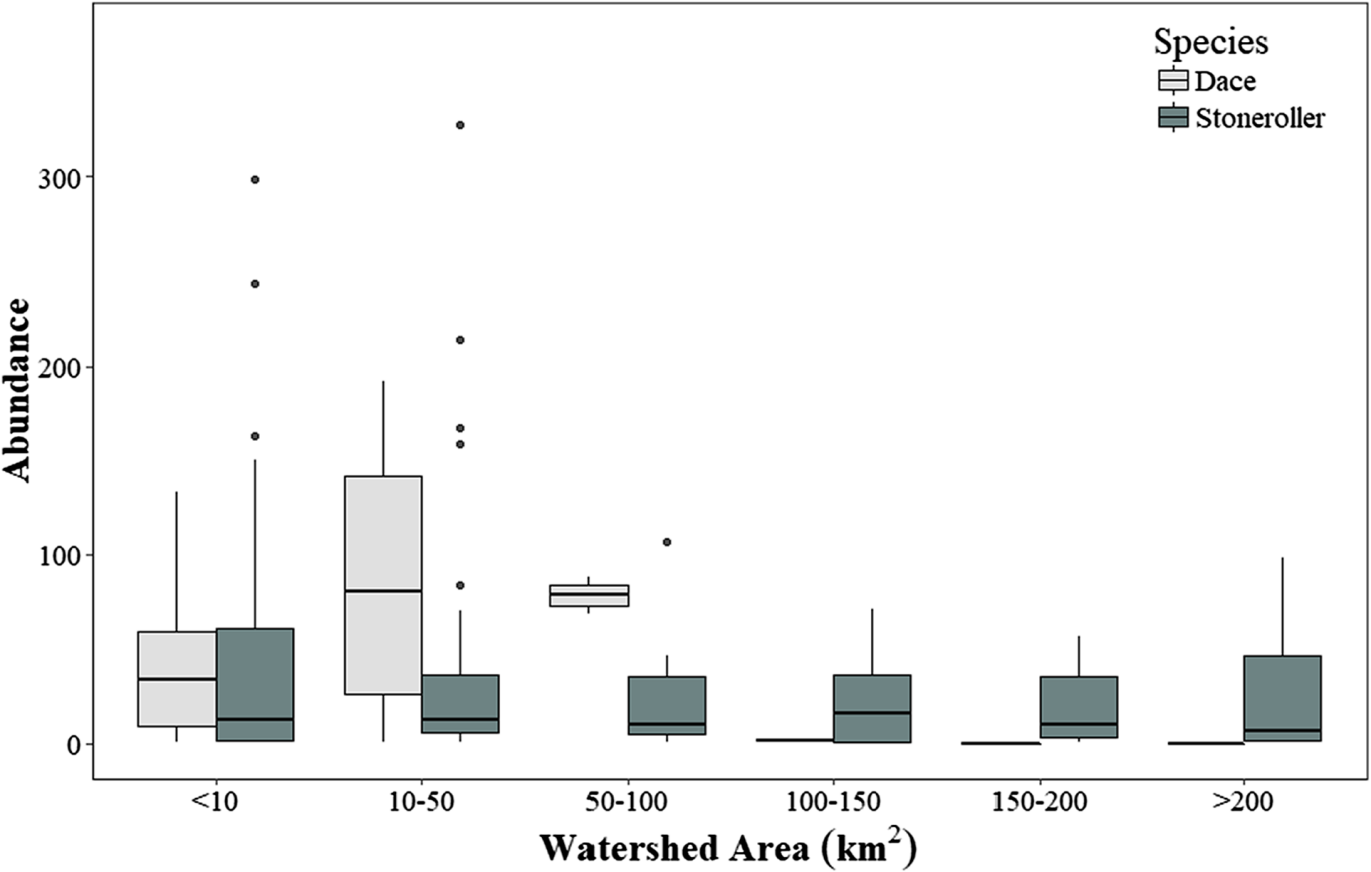
Boxplots representing abundances (count of individuals) of southern redbelly dace (light grey) and central stoneroller (dark grey) as a function of watershed area (km^2^). Boxes represent the median and the first and third quartile, and filled circles represent outliers. Dace populations occur more frequently in smaller watersheds, and stonerollers persist into larger watersheds.

Both dace and stonerollers were present and abundant in small streams (watershed areas <10–50 km^2^). Stoneroller populations remained abundant as stream size increased towards >200 km^2^, while populations of dace did not persist or were scarcely abundant (Fig. 1).

### Stream temperatures

**Figure 2:**
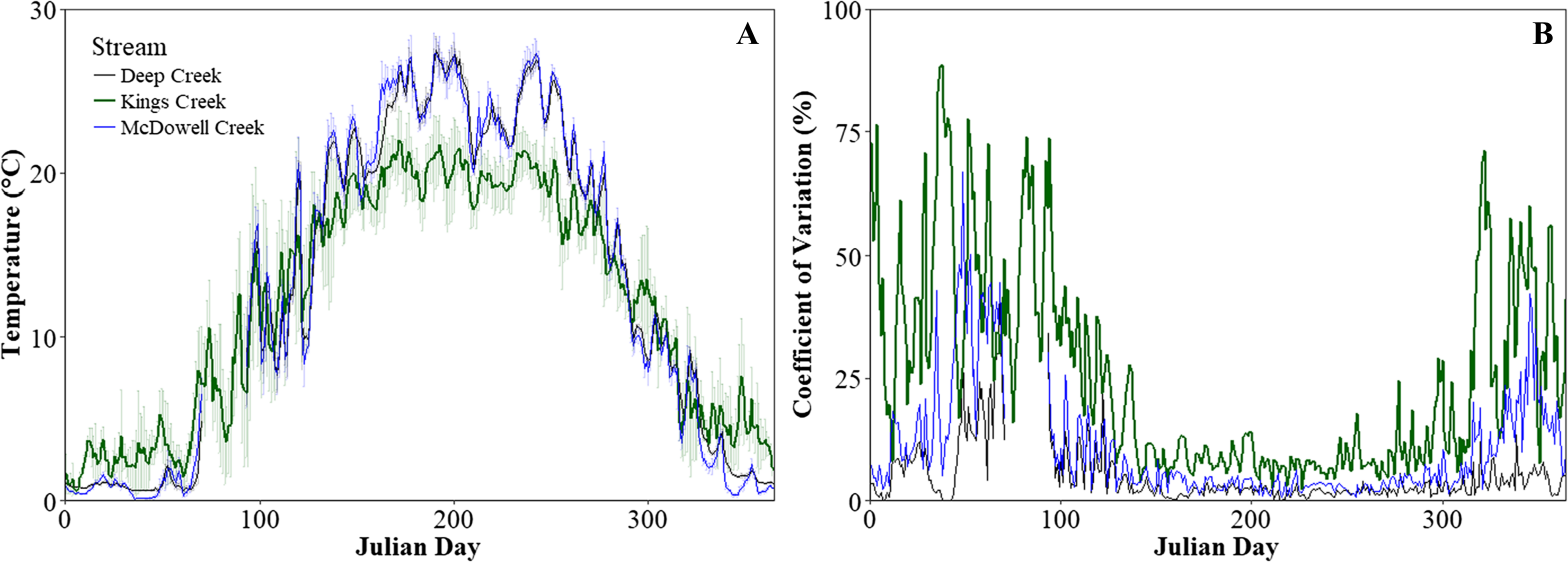
Mean (±1 SD) daily temperature (°C; A) and coefficient of variation (%; B) plotted for Julian day for three prairie streams in Kansas River drainage of the Flint Hills region (USA). Kings Creek (green) is a headwater reach that contains dace and stoneroller. Kings Creek feeds McDowell Creek (blue), which contains stonerollers but not dace. Deep Creek (black) is in the same river basin, is similar in size to McDowell Creek and contains stoneroller but not dace. Temperature data are from 2013 to 2014.

We calculated the mean daily temperatures (±1 SD) and the coefficient of variation (%) for daily temperature for three streams in the Kansas River basin. Kings Creek was ~5°C cooler, on average, during summer months compared to the more intermediate-sized McDowell and Deep Creeks (Fig. 2A). Additionally, temperatures in Kings Creek were typically more variable (Fig. 2B). Dace and stoneroller co-occur in Kings Creek. Whereas stoneroller occurs in both McDowell Creek and Deep Creek, dace are absent or very rare in these streams.

### CTM

**Figure 3:**
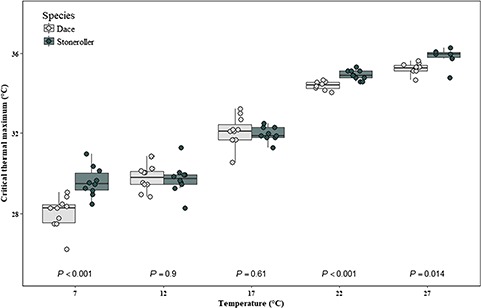
CTMs (°C) of southern redbelly dace (light grey) and central stoneroller (dark grey) acclimated to constant temperatures (°C) along a gradient in the laboratory. Boxes represent the median and the first and third quartile. *P*-values represent between-species post hoc comparisons (independent samples *t*-tests) and are considered significant at *P* ≤ 0.01.

There was a significant temperature × species interaction in the CTM of dace and stonerollers acclimated along a temperature gradient, after controlling for the effect of body size (Fig. 3; *F*_4,83_ = 6.725, *P* < 0.001). The covariate, body size, was significantly related to CTM (*F*_1,83_ = 6.973, *P* = 0.01) and regardless of species, larger fish had typically had slightly higher CTM (0.5–1°C) than did smaller fish. Not surprisingly, acclimation temperature had the strongest effect on CTM for both species, with dace CTM increasing by ~7°C and stoneroller CTM increasing by ~6°C between the coldest and warmest acclimation temperatures. Post hoc independent *t*-tests indicated that interspecific differences were less pronounced within each temperature treatment. While CTM was similar at intermediate temperatures (12°C, 17°C), stonerollers CTM was ~0.5–1.5°C higher than dace at low (7°C) and high (22°C, 27°C) acclimation temperatures (Fig. 3).

### Oxygen uptake and AMS

**Figure 4:**
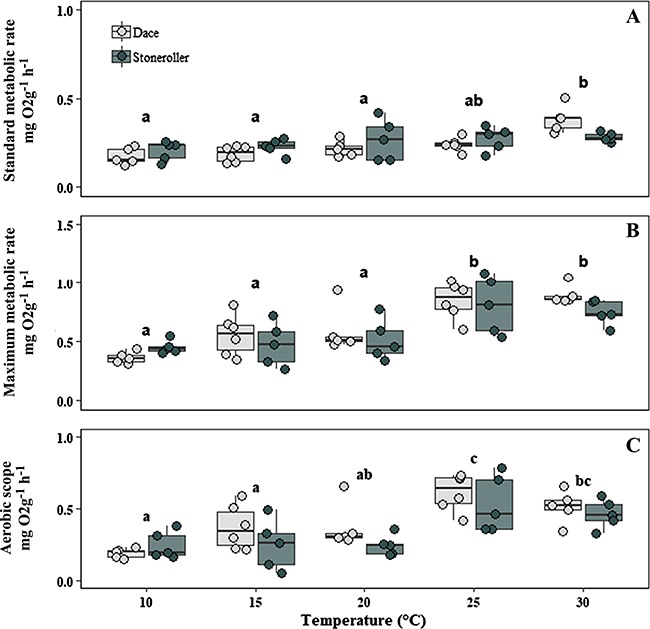
Mass-adjusted (A) SMR (mg O_2_ g^−1^ h^−1^), (B) MMR and (C) AMS of southern redbelly dace (light grey) and central stoneroller (dark grey) acclimated to constant temperature (°C) along a gradient in the laboratory. Filled circles represent individual data points. Boxes represent the median and the first and third quartile. Lowercase letters (a–c) represent significant differences between temperature treatments.

SMR, MMR and AMS did not differ between dace and stonerollers (SMR: *F*_1,46_ = 0.87, *P* = 0.356; MMR: *F*_1,46_ = 1.225, *P* = 0.274; AMS: *F*_1,46_ = 3.67, *P* = 0.062), but all metrics were significantly affected by acclimation temperature (SMR: *F*_4,46_ = 7.465, *P* < 0.001; MMR: *F*_4,46_ = 15.342, *P* < 0.001; AMS: *F*_4,46_ = 10.467, *P* < 0.001). Tukey post hoc tests revealed that mean values for SMR were significantly higher at 30°C than at 10, 15 and 20°C (Fig. 4A), while mean values for MMR were significantly higher at 25 and 30°C than other acclimation temperatures (Fig. 4B). Mean values for AMS were significantly higher at 25°C compared to the 10, 15 and 20°C treatments, while AMS at 30°C was significantly higher than the 10 and 15°C treatments (Fig. 4C).

### Critical swimming velocity

**Figure 5:**
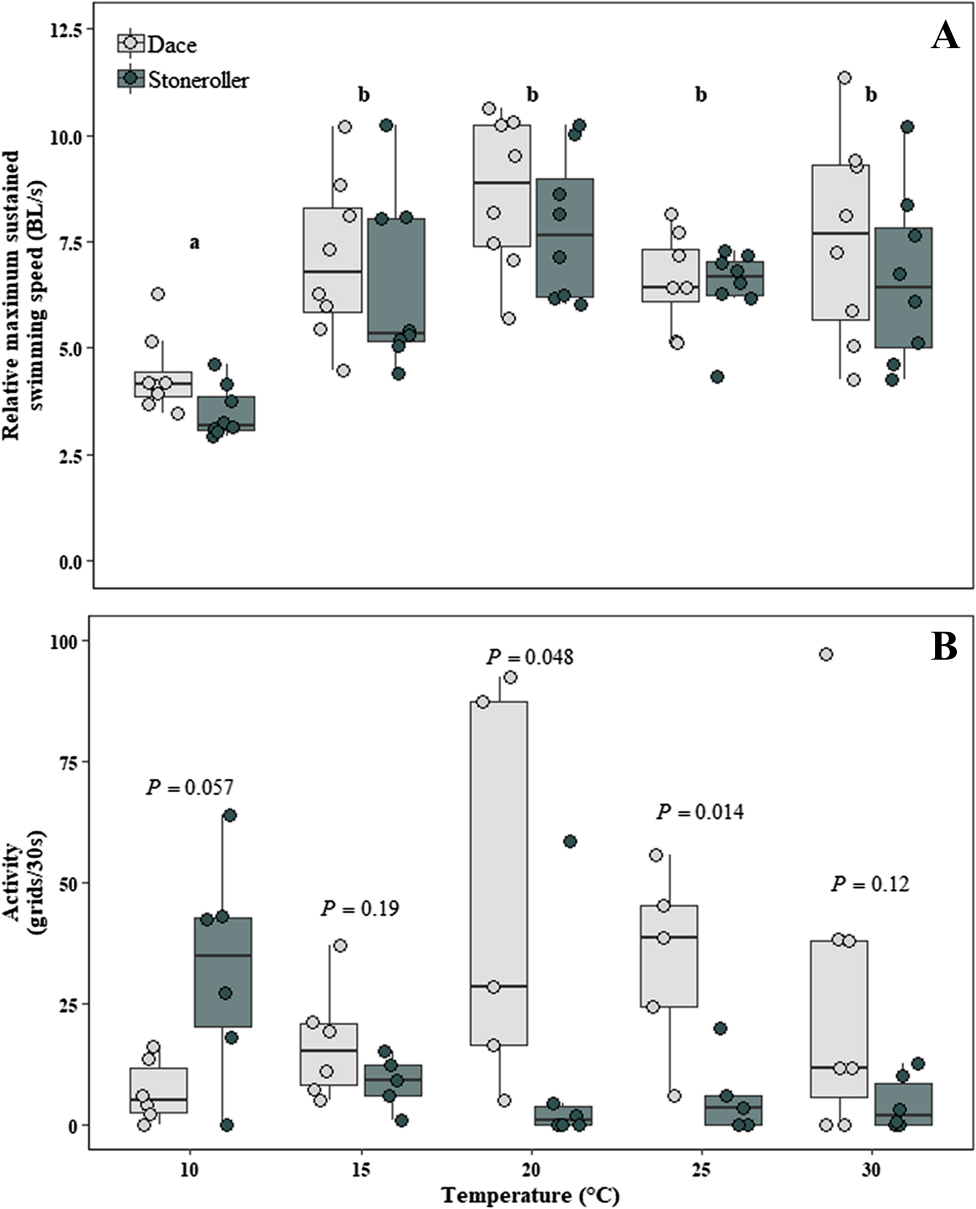
(A) Relative maximum sustained swimming speed (RU_crit_; BL/s) and (B) activity (the average number of 2 cm × 2 cm grids crossed in 30 s; grids/30 s) of southern redbelly dace (light grey) and central stoneroller (dark grey) acclimated to constant temperature (°C) along a gradient in the laboratory. Filled circles represent individual data points. Boxes represent the median and the first and third quartile. *P*-values represent between-species post hoc comparisons (independent samples *t*-tests) and are considered significant at *P* ≤ 0.01.

We detected significant effects of temperature (*F*_1,74_ = 21.658, *P* < 0.001) and species (*F*_1,74_ = 4.434, *P* = 0.039) on *RU*_crit_. Tukey post hoc tests revealed that mean values for *RU*_crit_ were significantly lower at 10°C than other acclimation temperatures (Fig. 5A). Dace typically had higher mean *RU*_crit_ than stoneroller, regardless of acclimation temperature treatment (Fig. 5A).

### Activity

Two-way ANOVA indicated a significant temperature × species interaction for activity (*F*_4,47_ = 3.991, *P* = 0.007). Post hoc independent *t*-tests indicated that interspecific differences were less pronounced within each temperature treatment. Dace activity increased towards intermediate temperatures before declining at high temperatures, while stoneroller activity was high at the lowest temperature and declined in warmer treatments (Fig. 5B).

## Discussion

While stonerollers persist and are abundant across a larger gradient of stream sizes throughout the Flint Hills of Kansas, dace appear to be more restricted to smaller sized streams (Fig. 1). Local stream temperature data indicate that smaller headwater streams in this region are typically cooler on average, particularly during the summer (Fig. 2)—and studies modelling stream temperature in the Flint Hills support this (Troia and Gido, 2014; Troia *et al*., 2016). We performed a comparative analysis of a series of traits associated with the thermal physiology of stonerollers and dace and found some subtle differences, but the direction and magnitude of these interspecific differences did not always align with our prediction that the measured responses of stonerollers for these traits would be higher than dace at warmer water temperatures. For example, stonerollers exhibited significantly higher CTMs than dace when acclimated to a warm (22°C) temperature, but there was no difference between species at the warmest (27 °C) temperature—although stonerollers trended towards higher CTM than dace at this temperature (Fig. 3) This provides some evidence that stonerollers might buffer thermal stress better than dace, and if our stream temperature data are indeed representative (Fig. 2) then stonerollers appear to have higher CTMs than dace under summer temperature conditions that are more typical of intermediate-sized streams. The range of CTM values we observed across acclimation treatments align well with what is already reported for both species under natural conditions (Scott, 1987; Mundahl, 1990). However, the lack of difference in CTMs between species at some acclimation temperatures suggests thermal tolerance alone is likely not a strong predictor of species distribution.

The greatest between-species contrast was the interaction between species and temperature for activity, where dace activity increased with temperature while the activity of stonerollers declined—a pattern that was counter to our predictions. The interaction in activity we observed between temperature and species indicates that temperature might affect the behaviour of these species differently or that spontaneous activity may trade off with other physiological functions not measured herein. Alternatively, the lower spontaneous activity of the stoneroller could be the result of the benthic-dwelling stoneroller maintaining a less active lifestyle compared to the dace, which typically inhabits the water column. Additionally, dace typically exhibited higher *U*_crit_ values compared to stonerollers, regardless of acclimation temperature, indicating that they are stronger swimmers overall. Hydrology can be an important driver of interspecific differences in swimming performance along a stream-size gradient in other systems. Higher sustained swimming performance has been observed in headwater salmonids in Japanese streams (Morita *et al*., 2016) and in Australian blackfishes (Allen-Ankins and Stoffels, 2017) when compared to a downstream species. Since both temperature and hydrology vary along gradients of stream size, this emphasizes the importance of considering the influence of both variables on species distributions due to the covarying nature of these habitat conditions along the river continuum.

Why do dace not occur in larger streams where stonerollers persist? Aerobic metabolism and swimming performances of dace and stonerollers did not differ at high temperatures indicating that these species are similar in their fundamental thermal niche along these axes, yet dace are typically absent from streams that regularly experience these high temperatures. While stonerollers could have a higher capacity to buffer thermal stress than dace in intermediate streams under summer-like thermal conditions, other ecological factors may also contribute to differences in the observed distributions of dace and stonerollers. For example, in grassland streams, more predatory species of fish occur downstream compared to headwaters, and the interaction between predation and competition may contribute to the exclusion of dace from larger, downstream reaches (Schlosser, 1982; Chase *et al*., 2002; Chesson and Kuang, 2008). Furthermore, relatively little is known regarding the role of temperature in mediating the outcomes of ecological interactions like competition and predation (Öhlund *et al*., 2015; Rogers *et al*., 2018). While an organism’s fundamental thermal niche is an important driver of distributional patterns, the outcome of ecological interactions (both independent of and influenced by temperature) may drive distributions through shaping of the realized thermal niche (Öhlund *et al*., 2015; Allen-Ankins and Stoffels, 2017).

Emerging evidence suggests that subtle, interacting characteristics among closely related and ecologically similar species may drive differences in ecological success (Dupoué *et al*., 2017; Porreca *et al*., 2017). We found some evidence that dace and stonerollers differ in components of their thermal biology but that the fundamental thermal niche of both species is very similar, at least for the metrics we examined. Low sample sizes in some of our experiments could have consequences on these results. High individual variability in physiological metrics may have contributed to the lack of differences we observed between species (e.g. Fig. 4), and a larger sample of fish tested may have better teased out differences if any do exist. Other ecological factors, such as hydrology, habitat heterogeneity and biotic interactions could be more important drivers of the distributions of these species than temperature or may interact with temperature and ultimately affect the realized thermal niche of these species. We addressed several mechanisms relating to the thermal niche of these species, which could potentially influence the distributional patterns we observe. However, other aspects of the life history of these organisms, such as growth or reproduction, could also be important components of their thermal niches and may differ between species. Our experiments also were conducted using adult fish and the influence of temperature on the early life history of these species is not well understood yet could be an important contributor to differences in growth and survival. Furthermore, the results of ecological interactions between these species could change as a function of temperature. Future research should continue to integrate the interactive effects of temperature and other environmental conditions on organismal, population and community level processes.

Finally, many laboratory studies are limited in that they use chronic exposures to constant temperatures to assess responses in physiological traits, which may not reflect how organisms respond to temperature change and variation in natural environments (Schulte *et al*., 2011). Recent work on thermal tolerance in zebrafish (Schaefer and Ryan, 2006) and Atlantic salmon (Corey *et al*., 2017) suggests that exposure to variable thermal regimes increases the thermal limits of these species compared to exposure to chronic temperatures. In grassland streams, intermediate reaches are typically warmer and more stable compared to smaller headwater reaches (Fig. 2). Incorporating thermal variability into tests of organismal responses to temperature might help elucidate how organisms respond to temperature in variable environments (Schulte *et al*., 2011; Drake *et al*., 2017). The increased frequency and severity of drought in prairie streams will likely exacerbated stream drying and potentially eliminate spring refugia (Dodds *et al*., 2004). For the dace, a species typical of small headwaters but absent downstream, the loss of these important thermal refugia may lead to local extirpation in some systems and contribute to conservation challenges. Understanding how organisms like dace, which may rely on conditions specific to these headwaters, respond along environmental gradients will be an important step in forecasting their response to anthropogenic change.
